# The miR-200 family in ovarian cancer

**DOI:** 10.18632/oncotarget.18343

**Published:** 2017-06-02

**Authors:** Maria Koutsaki, Massimo Libra, Demetrios A. Spandidos, Apostolos Zaravinos

**Affiliations:** ^1^ 3rd Department of Pediatrics of the National and Kapodistrian University of Athens, Attikon University Hospital, 12462 Athens, Greece; ^2^ Department of Biomedical and Biotechnological Sciences, Laboratory of Translational Oncology and Functional Genomics, Section of General and Clinical Pathology and Oncology, University of Catania, 95124 Catania, Italy; ^3^ Laboratory of Virology, Medical School, University of Crete, 71110 Heraklion, Crete, Greece; ^4^ Department of Life Sciences, School of Sciences, European University Cyprus, 1516 Nicosia, Cyprus

**Keywords:** miR-200 family, ovarian cancer, prognosis, disease stage, tumor histology

## Abstract

Ovarian cancer (OC) is the most lethal gynecological malignancy. Its insidious nature, manifesting with little to no symptoms until the disease progresses to metastasis, along with a wide diversity of histological subtypes and corresponding clinical behavior, poses significant therapeutic challenges. The genetic profiling of this aggressive tumor and its subtypes has led to the identification of various molecular markers of prognosis. Among these, the miR-200 family of miRNAs appears to play an important role. The deregulated expression of the miR-200 family members has been detected in a variety of OC studies. The present review examines the potential usefulness of the miR-200 family members as prognostic indicators in ovarian cancer and their impact across different OC publications, with a particular focus on prognostic features, such as disease stage, tumor histology, survival and response to chemotherapy. We present the potential usefulness of the miR-200 family genes as prognostic indicators in OC and highlight the tendency that miR-200 overexpression corresponds with an advanced cancer stage.

## INTRODUCTION

Ovarian cancer (OC) is the fifth more common type of cancer in the Western world [1, 2] and the leading cause of death due to gynecologic malignancy [3]. Its insidious nature, manifesting with little to no symptoms until the disease progresses to metastasis, along with a wide diversity of histological subtypes and corresponding clinical behavior, poses significant therapeutic challenges. Although the survival rate of patients with localized ovarian cancer (FIGO stages IA and IB) reaches 92%, early detection remains problematic, as the combined modalities (mostly serum markers and ultrasonographic screening) currently employed have limited effectiveness [4], as revealed by the relative overall survival rate of 45%, regardless of the disease stage [5]. Chemoresistance is another major feature of OC, with the majority of cases relapsing after cytoreduction and initial platinum-based chemotherapy [6].

Great progress has been made over the past decade in elucidating ovarian carcinogenetic mechanisms, beginning with the conceptualization of the dualistic model by Shih and Kurman [7]. Bridging the gap of heterogeneity in OC, the authors attempted to match the different histological subtypes to genetic changes in OC and proposed the presence of two main molecular tumorigenic pathways that lead to its occurrence: 1) a slow progressing pathway (type I tumors), including low-grade serous-papillary, endometrioid and borderline tumors of low malignant potential, and 2) a pathway with a more rapid course, mainly represented by high-grade serous carcinomas (type II tumors).

Given that OC is an aggressive gynecological malignancy, difficult to treat, with doubtful prior symptoms, it is a plausible candidate for genetic research. The miR-200 family is composed of 5 miRNA sequences: miR-200a, miR-200b, miR-200c, miR-141 and miR-429 [8], which, through the regulation of epithelial to mesenchymal transition (EMT), are fundamental in neoplastic transformation [9–12], and in organs with a high physiological turnover rate, such as the ovaries [13, 14]. Under this scope and following mounting publications linking the miR-200 family to OC progression, in this review, we attempt to present a critical review of the impact of the miR-200 family on OC, presenting information from various OC studies. We focus on the prognostic features of the disease, such as disease stage, tumor histology, patient survival and response to chemotherapy, with an aim to critically appraise the findings of various studies.

### Disease stage

As regards disease stage and metastatic potential, patients with OC are usually diagnosed at an advanced stage, with metastatic disease already present within the peritoneal cavity [15]. OC has a characteristic metastatic model, since it rarely metastasizes through the vasculature. Instead, it metastasizes either by direct extension from the ovarian tumor to neighboring organs (urinary bladder/colon) or through exfoliated tumor cells shedding into the peritoneal cavity and subsequent cancer cell seeding to distant abdominal sites [15]. Extensive seeding of the peritoneal cavity by tumor cells is often associated with ascites, particularly in advanced, high-grade serous carcinomas [15].

Cao et al. [16] investigated the association of the expression of miR-200 family members with the clinicopathological characteristics of patients with epithelial ovarian cancer (EOC). Their study involved 100 patients with EOC and 50 healthy subjects, and demonstrated that tumors with high a expression of miR-200a and miR-200b were more likely to be associated with an advanced stage and higher grade of the disease, while a high miR-200c expression was only significantly associated with an advanced stage of the disease [16]. The aberrant expression of these three miRNAs was also suggested to be associated with aggressive clinicopathological characteristics in patients with EOC [16]. Furthermore, Cittelly et al. [17] reported on miR-200c expression in an intraperitoneal xenograft model of human OC, and found that its restoration resulted in decreased tumor formation and tumor burden. In addition, Yang et al. [18] studied 10 primary serous ovarian tumors and 10 normal ovarian surface epithelium (OSE) cell lines, and reported that an increased miR-200a expression was associated with tumors of a high-grade and late-stage. The authors suggested that the deregulation of miR-200a may contribute to ovarian tumor progression rather than initiation, despite the small number of specimens. Interestingly, Xu et al. investigated miR-200a expression in EOC tumors and compared it to benign ovarian epithelial cysts and normal ovaries, and reported contradictory findings [19]. This group’s results demonstrated that the expression of miR-200a was significantly downregulated in late-stage disease (FIGO III–V and grade 3 groups) compared to early-stage (FIGO I–II and grade 1, 2 groups) tumors. Additionally, the authors found that relatively low miR-200a levels were observed in the “lymph node-positive” compared to the “lymph node-negative” group [19]. In a study on miR-200 family expression in serum, which included a relatively large number of patients with EOC (70 EOC samples vs 70 controls), Zuberi et al. [20] showed that elevated miR-200a and miR-200b expression levels were significantly associated with disease progression and in particular, miR-200a was associated with tumor stage. Additionally, the significant upregulation of miR-200c was found in patients with lymph node metastasis [20]. Further evidence was provided by Marchini et al. [21], who showed that miR-200c expression in patients with stage I EOC correlated with disease outcome. Ιn a blood cohort by Meng et al. [22] involving serum samples from 180 patients with EOC and 66 healthy women, the expression of miR-429 was found to be significantly upregulated in the EOC group. Additionally, miR-429 levels positively correlated with serum CA125 values and differed significantly between early-stage (FIGO I–II) and late-stage (FIGO III–IV) disease [22]. The same researchers, in a subgroup of 163 patients with EOC, demonstrated that circulating exosomal miR-200a, miR-200b and miR-200c concentrations were higher in patients with EOC than in healthy women [23]. Moreover, while the levels of miR-200a were found to be increased in all FIGO/lymph node stages, the levels of miR-200b and miR-200c were higher in patients with late-stage disease (FIGO III–IV) and with lymph node metastasis, than in those with early-stage (FIGO I–II) disease, suggesting that these exosomal miRNAs could discriminate between patients with EOC and healthy women, as well as between malignant and benign ovarian tumors [23]. The researchers concluded that the increased levels of exosomal miR-200b and miR-200c were associated with lymph node metastasis, an advanced FIGO stage (III–IV), the tumor marker, CA125, and with a poor overall survival, demonstrating that these miRNAs may be involved in tumor progression [23].

### Tumor histology

As regards tumor histology, Iorio et al. [24] were the first to report the possibility of “histotype-specific” miRNAs in OC. Their study involved 69 cases of epithelial OC of various histotypes and 15 normal ovaries. The authors described a miRNA expression signature that discriminated OC tissues from normal ovaries. Among the most overexpressed miRNAs within the signature were miR-200a, miR-200b, miR-200c and miR-141 [24]. When correlated to the clinicopathological tumor features, the miRNA expression profiles were found to differ in distinct OC histotypes. In particular, miR-200a and miR-200c were overexpressed in serous, endometrioid and clear cell histotypes, whereas miR-200b and miR-141 overexpression was only detected in endometrioid and serous histotypes. This upregulation was suggested to be due to the amplification of the miRNA genes [25]. The authors further commented that different OC histotypes presumably represent biologically and pathogenetically distinct entities of EOCs, although they are currently treated with identical therapeutic strategies [26]. Microarray analysis has confirmed that different histotypes (serous, mucinous, endometrioid and clear cell) show alterations of different pathways, probably reflecting the gene expression pattern of the organ of origin (fallopian tubes, colonic mucosa and endometrium) [26]. Another study by Wyman et al. [27] involving serous, clear cell and endometrioid EOC tumors, were compared to a normal human ovarian surface epithelium (HOSE) cell line. Using massive parallel sequencing, 8 miRNAs were identified as most overexpressed in OC tissue, including miR-200a, miR-200b and miR-200c. While all 8 miRNAs were found to be over-expressed in serous and clear cell cancer subtypes, miR-200(a/b/c) was found to be consistenty up-regulated in the endometrioid subtype as well. The authors considered a competitive advantage of their study, the fact that they used primary cultures of HOSE as the normal control group, instead of whole ovary tissue. On the basis that OC has an epithelial origin [28, 29], performing comparisons of OC tissue to an appropriate normal comparison group is a challenging problem in OC research. The monolayer ovarian surface epithelium comprises less than 1% of the cellular content of the whole ovary [29], and the use of whole ovary tissue inevitably leads to study bias deriving from sample contamination by nearby stromal cells. A serum cohort conducted by Zuberi et al. [20], involved blood serum samples from 70 patients with EOC of various histotypes compared to 70 age-matched, cancer-free female volunteers as controls. Using qRT-PCR, the researchers showed that miR-200a overexpression was strongly associated with the tumor histological subtype. Importantly, the authors found that miR-200a expression was significantly upregulated in the mucinous subtype of EOC.

The above-mentioned studies demonstrate the possibility that distinct miR-200 family member (mainly miR-200a and miR-200c) expression signatures are able to discriminate between OC histological subtypes (Table 1). Histotype-predictive miRNAs belonging to the miR-200 family have been reported in other solid malignancies, such as renal cancer. Working on renal cell tumors, Silva-Santos et al. [30] reported that th expression analysis of miR-141 or miR-200b could accurately distinguish renal cell tumors from normal renal tissues, and even between histological subtypes (oncocytoma, clear cell, papillary).

**Table 1:** Genomic OC studies focusing on the expression of miRNA-200 family members in relation to disease prognostic features

Study (Year)	Genes	Study groups	Prognostic Feature	Results-Comments
Iorio et al. (2007) [20]	miR-200a, miR-200b, miR-200c, miR-141	Normal ovarian tissues (*n =* 15) vs EOC tissue samples (*n =* 69)	Tumor histology	1) miR-200a and miR-200c are upregulated in serous, endometrioid, clear cell type. 2) miR-200b and miR-141 are upregulated in serous, endometrioid type.
Yang et al. (2008) [14]	miR-200a	HOSE cell lines (*n =* 10) vs primary serous OC tumors (*n =* 10)	Disease stage	miR-200a up-regulation is associated with late-stage and high-grade disease
Nam et al. (2008) [50]	miR-200a, miR-200b, miR-200c, miR-141, miR-429	Serous OC tissue samples (*n =* 20) vs normal ovary tissues (*n =* 20)	Survival	Increased expression of all miR-200 family members significantly correlates with decreased progression-free and overall survival.
Cochrane et al. (2009) [37]	miR-200c	OC cell lines (Hec50, AN3CA) vs immortalized OSE cells	Chemotherapy Response	1) Decreased miR-200c expression promotes invasion. 2) Restoration of miR-200c expression restores E-cadherin expression and reduces migration and invasion. 3) Restoration of miR-200c expression enhances chemosensitivity to microtubule-targeting agents by 85%.
Hu et al. (2009) [52]	miR-200a, miR-200b, miR-429	55 advanced OC tumors	Survival	1) High miR-200a expression is associated with an improved overall survival and reduced cancer recurrence. 2) A miR-200a expression signature is a robust predictor of disease outcome. 2) This miR-200a expression signature is independent of other clinicopathological features. 4) Overexpression of the miR-200b-429 cluster inhibits ovarian cancer cell migration.
Wyman et al. (2009) [23]	miR-200a, miR-200b, miR-200c, miR-141, miR-429	HOSE cells vs OC tumors (19 serous, 4 clear cell, 10 endometrioid)	Tumor histology	miR-200a, miR-200b, miR-200c are upregulated in all 3 subtypes of OC.
Leskelä et al. (2011) [39]	miR-200a, miR-200b, miR-200c, miR-141, miR-429	ovarian carcinoma tumors (*n =* 72)	Survival Chemotherapy Response	1) Low miR-200c, miR-429 and miR-141 exhibits a trend towards a poor PFS. 2) Low miR-200c expression is associated with disease recurrence and reduced OS. 3) Low miR-200c expression is associated with high tumoral β-tubulin III expression, and thus increased resistance to paclitaxel-based therapies.
Marchini et al. (2011) [17]	miR-200b, miR-200c	EOC stage I tumors (*n =* 144)	Survival	1) miR-200c expression is downregulated in disease relapsers compared to non-relapsers. 2) Upregulation of miR-200c is associated with an improved PFS and OS. 3) Upregulation of miR-200c is an independent prognostic factor of favorable survival, regardless of clinicopathological covariates.
Mateescu et al. (2011) [38]	miR-200a, miR-141	Mouse model	Survival Chemotherapy Response	1) Enhanced expression of both genes increases tumor growth. 2) miR-200a overexpression is associated with an oxidative stress-dependent signature which correlates with an improved survival due to treatment responsiveness.
Cittelly et al. (2012) [13]	miR-200c	Various OC cell lines	Survival Chemotherapy Response	miR-200c restoration in established tumors delays tumor progression and increases sensitivity to paclitaxel .
Prislei et al. (2013) [40]	miR-200c	OC tumors (*n =* 220) OC cell lines (ovarian adenocarcinoma derived lines)	Survival Chemotherapy Response	1) Stable overexpression of miR-200c is observed in chemoresistant cell lines compared to chemosensitive lines, regardless of whether chemoresistance was inherent or acquired. 2) Positive correlation between miR-200c and TUBB3 expression. 3) miR-200c overexpression correlated with good or poor survival of patients with OC, depending on the cellular localization of HuR protein.
Pecot et al. (2013) [58]	miR-200a, miR-200b, miR-200c, miR-141, miR-429	ovarian adenocarcinomas (*n =* 579) OC metastatic cell line (HeyA8)	Survival	1) Low expression of miR-200c is significantly associated with a worse overall survival. 2) Nanoparticle delivery of miR-200a and miR-200b in HeyA8 metastatic cells resulted in a 92% reduction in metastatic burden as compared with control miRNA, while improving vasculature organization.
van Jaarsveld et al. (2013) [41]	miR-200c, miR-141	OC cell lines (cisplatin-sensitive vs cisplatin-resistant) 132 primary OC tumors (108 serous, 24 non-serous)	Chemotherapy Response	1) Overexpression of miR-141 results in enhanced resistance to cisplatin in OC cell lines. 2) miR-141 expression levels are higher in primary non-serous OC tumors that did not respond well to therapy.
Wang et al. (2014) [42]	miR-429	Metastatic OC cell line (OCI-984)	Chemotherapy Response	OCI-984 cells transfected with miR-429 exhibited morphological and functional features consistent with MET, resulting in a concomitant significant increase in the sensitivity of the converted cells to cisplatin.
Wang et al. (2014) [59]	miR-200a	Primary serous OC tumors (*n =* 56) vs benign ovarian lesions (*n =* 30)	Survival	Patients with a low miR-200a expression have higher OS rates.
Vilming Elgaaen et al. (2014) [60]	miR-200a, miR-200b, miR-200c, miR-141	OC tumors (HGSC *n =* 12, CCC *n =* 9) vs normal OSE samples (*n =* 9)	Survival	High miR-200c expression is associated with a poor PFS and OS in the HGSC group.
Cao et al. (2014) [12]	miR-200a, miR-200b, miR-200c, miR-141, miR-429	EOC tissue samples (*n =* 100) vs OSE tissues (*n =* 50)	Disease stage Survival	1) Overexpression of miR-200a, miR-200b and miR-200c correlates with a shorter OS in patients with EOC. 2) Tumors with a high miR-200a and miR-200b expression are both more likely to be associated with an advanced stage and higher grade. 3) miR-200c expression is significantly associated with advanced-stage disease.
Xu et al. (2014) [15]	miR-200a	EOC tumors (*n =* 62) vs benign epithelial lessions (*n =* 24) and normal ovaries (*n =* 25)	Disease stage	1) Expression of miR-200a is significantly downregulated in late-stage disease (FIGO III-V and grade 3 groups) compared with early-stage (FIGO I-II and grade 1, 2 groups) tumors. 2) The miR-200a expression levels in EOC tissues with lymph node metastasis are reduced compared to those without lymph node metastasis.
Zuberi et al. (2015) [16]	miR-200a, miR-200b, miR-200c	Serum samples from: EOC patients (*n =* 70) vs healthy female age-matched volunteers (*n =* 70)	Survival Disease stage	1) Expression levels of miR-200a and miR-200c in serum from patients with EOC are significantly associated with disease progression. 2) miR-200a overexpression is associated with tumor stage. 3) miR-200c is upregulated in patients with lymph node metastasis.
Brozovic et al. (2015) [43]	miR-200a, miR-200b, miR-200c, miR-141, miR-429	Human OC cell lines, paclitaxel-sensitive and paclitaxel-resistant	Chemotherapy Response	1) Decreased expression of all miR-200 family members in paclitaxel-resistant cells. 2) Significant decrease in the expression of miR-200c and miR-141 in paclitaxel-resistant cells. 3) Retroviral transfection of both cell lines with miR-200 mimics, results in increased resistance to carboplatin after miR-c141 cluster transfection, but not after transfection with the miR-ab429 cluster. 4) Inhibition of miR-200c and miR-141 results in the upregulation of TUBB3 in paclitaxel-sensitive cell lines. 5) miR-200c-141 cluster treatment of the paclitaxel-resistant cells, although conferring resistance to carboplatin, sensitized the cells to paclitaxel.
Meng et al. (2015) [18]	miR-429	Serum samples from: EOC patients (*n =* 180) vs healthy women (*n =* 66)	Survival Disease stage	1) Serum levels of miR-429 positively correlate with serum CA-125 values and disease stage. 2) miR-429 is an independent predictor of OS.
Meng et al. (2016) [19]	miR-200a, miR-200b, miR-200c	Serum samples from: EOC patients (*n =* 163) vs benign ovarian disease (*n =* 20) and healthy women (*n =* 32)	Survival Disease stage	1) miR-200b and miR-200c expression levels are higher in patients with late-stage disease (FIGO III–IV) with lymph node metastasis, than in those with early-stage (FIGO I–II) disease. 2) Exosomal miR-200a, miR-200b and miR-200c could discriminate between patients with EOC and healthy women, and between malignant and benign ovarian tumors. 3) Increased miR-200b and miR-200c expression in EOC is associated with a shorter overall survival.
Bagnoli et al. (2016) [62]	miR-200a-3p, miR-200b-3p, miR-200c-3p, miR-141-3p, miR-429	Tumor biopsies of OC patients from two EOC clinical trials (*n =* 715) vs normal ovaries (*n =* 179)	Survival	1) A 35-microRNA-based prognostic model for predicting the risk of progression or relapse in OC (MiROvaR), including all miR-200 family members (miR-200a-3p, miR-200b-3p, miR-200c-3p, miR-141-3p and miR-429) allowed patient stratification into low- and high-risk groups. 2) All miR-200 members are downregulated in most of the patients classified by MiROvaR as being at high risk of relapse.
Calura et al. (2016) [61]	miR-200a-3p, miR-200b-3p, miR-200c-3p, miR-141-3p, miR-429	tumor biopsies from stage I EOC (*n =* 203)	Survival	All miR-200 family members are associated with OS and PFS, in stage I EOC tumors.

OC: Ovarian Cancer; EOC: Epithelial Ovarian Cancer; OSE: Ovarian Surface Epithelium; HOSE: Human Ovarian Surface Epithelium; HIOSE: Human Immortalized Ovarian Surface Epithelium; PFS: Progression-Free Survival; OS: Overall Survival; HGSC: High Grade Serous Carcinoma; CCC: Clear Cell Carcinoma; TUBB3: class III β-tubulin; MiROvaR: miRNA-based predictor of Risk of Ovarian Cancer Relapse or Progression.

In the past, miRNA expression profiling has been successfully used for classifying human cancers [25, 31, 32]. miRNAs have been proven to be surprisingly informative in reflecting the developmental lineage and differentiation state of poorly differentiated tumors [32]. A gene expression study showed that genes expressed in different ovarian carcinomas were concordantly expressed in the normal tissues they resemble histologically [26]. Three anatomical sites are the potential origin of high grade serous carcinomas: the ovarian surface epithelium, the fallopian tube epithelium and the mesothelium covering the surface of the peritoneal cavity [26]. Similarly, a plausible scenario regarding tumor histology and miR-200 family expression would be that distinct patterns of miRNA overexpression could represent the developmental ancestry of distinct ovarian tumors.

### Chemotherapy response

Despite the improvement in the management of OC over the past 30 years, with treatments such as cytoreductive (debulking) surgery and optimized combinational chemotherapy, usually with platinum-based drugs combined with taxanes, the overall cure rate is only 30%, mainly due to OC resistance to chemotherapy [33, 34]. In general, clear-cell carcinomas, which are associated with a worse prognosis in early-stage disease, and mucinous carcinomas do not respond as well as serous and endometrioid tumors to platinum- and taxane-based chemotherapy [35–37]. Original evidence regarding the association of miRNA expression and cancer chemosensitivity was firstly raised by Meng et al. in cholangiocarcinoma [38]. It was found that the inhibition of miR-21 and miR-200b enhanced the sensitivity of cholangiocarcinoma cells to gemcitabine, probably by the modulation of CLOCK, PTEN and PTPN12, and downstream oncogene products, such as c-Abl, Src and Ras [38, 39]. Furthermore, mounting evidence indicates the existence of similarities between drug-resistant and metastatic cancer cells in terms of resistance to anoikis and enhanced invasiveness [17, 40].

As regards the influence of the miR-200 family on the response to chemotherapy in OC, Cochrane et al. [41] demonstrated that the restoration of miR-200c in various ovarian cancerous cell cultures led to a marked decrease in cell migration and invasion, and to an up to 85% increase in sensitivity to microtubule-targeting chemotherapeutic agents, presumably through the downregulation of class III β-tubulin (TUBB3). Additionally, a low miR-200c expression strongly correlated with the lack of E-cadherin expression and the gain of mesenchymal markers, including ZEB1, while the restoration of miR-200c seemed to restore E-cadherin expression and reduce migration and invasion. Using mouse models, Mateescu et al. [42] showed that an enhanced miR-141 and miR-200a expression increased tumor growth, but at the same time rendered the tumor more susceptible to treatment. The authors suggested that since miR-141 and miR-200a both target p38α and modulate the oxidative stress response, their enhanced expression could mimic p38α deficiency and enhance chemosensitivity [42]. In accordance with previous studies are the findings of Leskelä et al. [43], who studied 72 cases of OC, and showed that a low tumoral miR-200 family content was significantly associated with a high β-tubulin III protein expression [43]. Additionally, in a panel of 57 advanced OC tumors, patients without a complete response had lower miR-200c levels than patients with a complete response. The authors indicated that a low miR-200c expression resulted in a high β-tubulin III expression, and thus increased resistance to paclitaxel-based therapies, and suggested that miR-200 downregulates β-tubulin III in ovarian tumors [43]. Similar findings were reported by Marchini et al. [21] in a series of 144 patients with stage I EOC, where the downregulation of miR-200c was observed in relapsers compared to non-relapsers. Studying OC cell lines overexpressing miR-200c, Cittelly et al. [17] demonstrated that the *in vitro* restoration of miR-200c in OC cells resulted in increased anoikis sensitivity and reduced adherence to components of the extracellular matrix. Additionally, the restoration of miR-200c in an intraperitoneal xenograft model of human OC resulted in decreased tumor formation and ultimately, tumor burden [17]. As previously shown, in established tumors where the restoration of miR-200c seems to delay tumor progression and increase sensitivity to paclitaxel, presumably by targeting the TUBB3 gene [41], Cittelly et al. [17] suggested that the restoration of miR-200c immediately before treatment could enhance the treatment response or allow for a lower initial dose. In a panel of ovarian adenocarcinoma cell lines, a direct correlation between miR200c overexpression and chemoresistance was also demonstrated by Prislei et al. [44]. The stable overexpression of miR-200c was noted in chemoresistant cell lines (A2780 and Hey), regardless of whether the resistance to paclitaxel and cisplatin was inherent or acquired.

The differential expression of miR-141 and miR-200c between cisplatin-sensitive and cisplatin-resistant OC cell lines was shown by van Jaarsveld et al. [45]. In their study, the overexpression of miR-141 resulted in enhanced resistance to cisplatin in OC cell lines. Additionally, in a setting of 132 primary ovarian tumors (108 serous and 24 non-serous), although no differences were observed in the serous tumors, miR-141 expression levels were higher in non-serous ovarian tumors that did not respond well to therapy [45]. The authors further demonstrated that miR-141 directly targets KEAP1 and that the downregulation of KEAP1 through miR-141 overexpression could induce cisplatin resistance, presumably through the activation of the NF-κB pathway.

Evidence regarding the role of miR-429 in the response to chemotherapy in OC was provided by Wang et al. [46]. The researchers generated a primary cell line deriving from free-floating OC ascites cells (OCI-984 cell line) and sought to determine whether metastasizing OC cells could be induced by miR-429 to undergo mesenchymal-to-epithelial transition (MET) and become sensitized to established first-line platinum-based therapies. Drug sensitivity assays showed that OCI-984 cells transfected with miR-429 exhibited morphological and functional features , as well as the “switching on” of epithelial markers consistent with MET, resulting in a concomitant significant increase in the sensitivity of the converted cells to cisplatin [46]. More recently, Brozovic et al. demonstrated that miR-200 family members differentially regulated sensitivity to paclitaxel and carboplatin in human OC cell lines [47]. Using two ovarian cancer cell lines, OVCAR-3 and MES-OV, and their paclitaxel-resistant variants, OVCAR-3/TP and MES-OV/TP, the authors showed a marked decrease in the expression of miR-200c and miR-141 in OVCAR-3/TP cells, while all five members of the miR-200 family were decreased in MES-OV/TP cells [47]. The retroviral transfection of both lines with miR-200 mimics resulted in a 4-fold increased resistance to carboplatin after miR-c141 cluster transfection, but not after transfection with the miR-ab429 cluster [47]. Additionally, miR-200c and miR-141 mimics treatment, although conferring resistance to carboplatin, sensitized MES-OV/TP cells to paclitaxel [47]. It is known that the expression of ZEB1, ZEB2 and SNAI2 inversely correlates with the expression of the miR-200 family [48–50]. In the study by Brozovic et al. [47], both resistant variants displayed a strong EMT phenotype, with a strong expression of ZEB1, ZEB2 and SNAI2 in miR-141 and miR-200c treated OVCAR-3 cells. The authors also examined the expression of TUBB3 and found that the inhibition of miR-200c and miR-141 resulted in the upregulation of this β-tubulin isotype in the paclitaxel-sensitive lines [47], verifying previous reports regarding the inverse correlation between miR-200 members and [41, 43] EMT markers.

### Survival

Survival in OC is limited, even with prompt diagnosis [51, 52] and it appears that, at best, the overall survival after original diagnosis does not exceed 5 years [2, 53]. Of the studies examining survival in OC and the impact of miR-200 family members, Nam et al. [54] were the first to investigate the prognostic value of these miRNAs. A higher expression of all miR-200 members (200a/b/c, 141, 429) was found to significantly correlate with decreased progression-free and overall survival [54]. This overexpression was hypothesized to be due to genetic amplification, as previously shown for miR-200a [25, 55], eventually rendering the tumor cells less susceptible to anti-neoplastic drugs [39]. By performing miRNA expression profiling analysis of 55 advanced ovarian tumors, Hu et al. showed that three miR-200 miRNAs (miR-200a, miR-200b and miR-429) in the miR-200b–429 cluster were significantly associated with cancer recurrence and overall survival [56]. In particular, a low miR-200a expression was significantly associated with recurrence and poor overall survival. The miR-200a expression signature was evaluated as highly predictive of recurrence-free and overall survival, suggesting that miR-200a is a robust predictor of disease outcome [56]. What is more, the miR-200a signature was independent of other clinicopathological features, maintaining its association with disease outcome, regardless of other clinical variables for outcome prediction [56]. Leskelä et al. found that miR-429 expression was significantly associated with recurrence-free survival and overall survival (OS) in patients with OC, and miR-200c and miR-141 were associated with recurrence-free survival [43]. As mentioned earlier, a low miR-200c expression was associated with a high tumoral β-tubulin III expression and thus increased resistance to paclitaxel-based therapies, justifying the poor progression-free survival and poor overall survival trends observed in this group. The authors concluded by suggesting that miR-200 members downregulated β-tubulin III in ovarian tumors [43]. Similar findings were reported in the study by Cittelly et al., where miR-200c restoration to established tumors delayed tumor progression and prolonged survival through the downregulation of TUBB3 [17]. A high tumoral β-tubulin III expression was shown to be associated with a worse survival in a similar study [57] and it is a well-known feature in other cancer types, such as non-small cell lung [58, 59], breast [60] and head and neck cancer [61]. Prislei et al. [44] went one step ahead, in suggesting a model for the combined regulatory activity of miR-200c and the regulatory RNA-binding protein HuR on TUBB3 expression in ovarian cancer. They demonstrated that the overexpression of miR-200c correlated with good or bad prognosis in OC, depending on the cellular localization of HuR. In particular, when HuR was nuclear, the high expression of miR-200c inhibited TUBB3 expression and resulted in a good prognosis, whereas when HuR was expressed in the cytoplasm, the same miRNA enhanced TUBB3 expression and led to a poor outcome [44]. In stage I EOC, Marchini et al. [21] demonstrated that miR-200c upregulation was associated with an improved progression-free survival and overall survival, independent of clinical covariates. The authors concluded by proposing that miR-200c expression was a predictor of disease outcome in early-stage EOC, as well as a candidate biomarker of relapsing disease [21]. In a mouse model of OC, Mateescu et al. [42] showed that miR-200a overexpression was associated with an oxidative stress-dependent signature which correlated with an improved survival due to treatment responsiveness. In a large retrospective cohort of adenocarcinomas including 579 ovarian samples, Pecot et al. showed that a low expression of miR-200c was significantly associated with a worse overall survival [62]. To assess the effects of the miR-200 family on angiogenesis, the authors used a metastatic HeyA8 model. The delivery of miR-200a and miR-200b resulted in a 92% reduction in metastatic burden as compared with the control miRNA, while the tumor vasculature following miR-200 treatment was significantly less permeable and the vessels were more organized and less tortuous than the control miRNA group [62]. The authors concluded by emphasizing that miR-200 members can have pronounced therapeutic effects on decreasing angiogenesis and metastasis formation in ovarian cancer, and that these findings could have important translational implications on the path to the clinical development of miRNA-based therapies [62]. Wang et al. compared 56 primary serous ovarian tumors to benign ovarian lesions and showed that patients exhibiting a low miR-200a expression had higher overall survival rates [63]. Furthermore, in patients with EOC, Cao et al. found that a high miR-200a, miR-200b and miR-200c expression significantly correlated with a shorter overall survival than the corresponding controls [16]. In a global miRNA expression profiling study, comparing high grade serous carcinomas (HGSC) and clear cell ovarian tumors with normal OSE samples, Elqaaen et al. showed that a high miR-200c-3p expression was associated with poor progression-free and overall survival in patients with HGSC, suggesting that miR-200c-3p is a candidate prognostic marker for high grade serous carcinoma [64]. Zuberi et al. focused their study on EPC patient-derived blood serum and showed that miR-200a and miR-200c expression was significantly associated with disease progression [20]. In a similar study on patients with EOC by Meng et al., miR-429 up-regulation was demonstrated to be an independent predictor of overall survival [23]. The same research group also found that an increased miR-200b and miR-200c expression in EOC was associated with a shorter overall survival [23]. Recently, Calura et al. [65] identified a prognostic cell pathway, composed of 16 miRNAs and 10 genes, that was associated with overall survival (OS) and progression-free survival (PFS) in stage I EOC tumors. Among these, miR-141-3p and miR-2000c-3p were significantly overexpressed. The authors verified Marchini›s et al. report about miR-200c-3p predictive power in EOC [21]. They further added that this prognostic signature could help stratify patients into risk classes [65]. Another prognostic model was designed by Bagnoli et al., who developed and validated a signature based on 35 miRNAs (miRNA-based predictor of Risk of Ovarian Cancer Relapse or progression, MiROvaR). Their signature was used for predicting the risk of progression or early relapse in EOC, also allowing patient stratification into low- and high-risk groups, in terms of cancer recurrence, with a difference in the median progression-free survival of 20 months between the high-risk and low-risk groups [66]. This miRNA signature maintained its independent prognostic effect when adjusted for relevant clinical covariates [66]. MiROvaR included all members of the miR-200 family (miR-200a-3p, miR-200b-3p, miR-200c-3p, miR-141-3p and miR-429) and it was demonstrated that all family members were down-regulated in most of the patients classified by MiROvaR as being at a high risk of relapse [66], further supporting the regulatory role of these miRNAs as inhibitors of EMT [48].

Overall, Table 1 summarizes the genomic studies focusing on the expression of the miRNA-200 family members in OC with regard to prognostic features, such as disease stage, tumor histology, survival and response to chemotherapy.

## DISCUSSION

As regards disease stage, with the exception of the study by Xu et al. [19], all four miR-200 members are unanimously overexpressed in late-stage OC disease compared to early-stage OC disease. Thus, miR-200 overexpression seems to correlate with disease progression, rather than initiation (as previously commented by Yang et al. [18]). Similar findings have been reported in other cancer types. For example, increased levels of miR-141, miR-200a and miR-200b were reported in patients with metastatic compared to non-metastatic colorectal cancer [67]. By contrast, according to Xu et al. [19], the expression of miR-200a was significantly downregulated in late-stage compared to early-stage EOC tumors. A similar expression pattern was observed for E-Cadherin, which is abundant in early-stage and low in late-stage tumors [19]. This could be explained on the basis of EMT induction in the early stages of the carcinogenesis, when malignant cells undergo EMT in order to acquire an invasive phenotype. When cells reach their metastatic sites, they tend to lose their invasive characteristics and re-epithelialization occurs. Thus, late-stage disease is characterized by low EMT, with concurrently low miR-200a and E-cadherin levels. This hypothesis could also partially explain the low miR-200a expression in the lymph node-positive, compared to the lymph node-negative group, irrespective of disease stage [19]. The lymph node-positive group could possibly represent re-epithelialization sites within the nodes, consistent with absent EMT. This is further supported by the finding of a high miR-200a and E-cadherin expression in benign ovarian tumors [19]. These are generally considered hyper-proliferative lesions, rather than malignancies, therefore a high cellular turn-over and active EMT would be expected.

A regards tumor histology, distinct expression patterns of the miR-200 members could discriminate between OC histological subtypes. Iorio et al. [24] found the upregulation of miR-200a and miR-200c in all 3 basic ovarian carcinoma histological subtypes (serous, endometrioid and clear cell), while miR-200b and miR-141 were upregulated only in the serous and endometrioid type. The possibility that the miR-200 family can be used to distinguish between histological subtypes was also reported in renal cancer. For example, Silva-Santos et al. found that miR-141 or miR-200b could accurately distinguish renal cell tumors from normal renal tissues, and even between histological subtypes, based on their expression profiles [30]. miR-141 differential expression could successfully discriminate between estrogen-dependent and estrogen-independent endometrial adenocarcinoma subtypes [68]. These findings provide beneficial evidence for the microRNA-200c-141 cluster to be viewed as a candidate serum biomarker of the histological discrimination of ovarian tumors into clinically relevant subgroups.

Patient survival studies have, without a doubt, attracted a major scientific interest. What is more, specific miR-200 expression signatures have been integrated to create prognostic models [65, 66]. As a general comment for all miR-200 members, it seems that there is a trend for an increased expression which correlates with a poor overall survival (Table 1). As regards miR-200a, its overexpression seems to be related with a poor [16, 20, 54, 63] rather than with a favorable overall survival [42, 56, 62]. Hu et al. [56] reported that the miR-200a expression signature was an independent prognostic factor, irrespective of other clinicopathological features. Similarly, miR-200b overexpression seems to correlate with a shorter, rather than a prolonged overall survival [16, 23, 54, 56, 62]. As regards miR-200c, its increased expression seems to correspond with a decreased overall survival [16, 20, 23, 54, 64]. This occurs presumably through the upregulation of EMT. Hurteau et al. [69] found that the overexpression of the microRNA miR-200c led to the reduced expression of transcription factor 8, and thus to an increased expression of E-cadherin, which induced EMT. As regards miR-141, Leskela et al. [43] reported that alow miR-141 exhibited a trend towards a poor progression-free survival, while Nam et al. [54] demonstrated opposite results (Table 1).

In colorectal cancer (CRC), in a blood cohort study by Maierthaler et al. [67], increased serum levels of miR-141 in were found in patients with metastatic compared to non-metastatic disease. Cheng et al. [70] reported that high plasma levels of miR-141 were significantly associated with a poor survival in patients with metastatic CRC in two independent sample cohorts.

The trend of an increased expression of miR-200 family members corresponding to a poor survival has been verified in other cancer types. In breast cancer, Madhavan et al. [71] reported that increased serum levels of all 5 miR-200 family members were predictive of a poor OS. Furthermore, increased levels of miR-200a, miR-200b, miR-200c in plasma samples of former breast cancer patients could indicate metastasis up to two years prior to clinical diagnosis [71]. In addition, in breast cancer, Raychaudhuri et al. [72] found that a low expression of miR-200c was associated with an increased overall survival. In a meta-analysis of the prognostic role of miR-200c-141 cluster in various human solid malignant neoplasms, the high expression of both miR-200c and miR-141 in serum, but not in tissues, was found to be associated with a poor OS [73]. By contrast, Maierthaler et al. [67] found low levels of circulating miR-200b and 200c to be associated with a “bad” prognosis subtype in patients with colorectal cancer. Another remark that could partially explain the discrepancy in the pattern of expression among miR-200 family members in OC, particularly for the blood serum studies, is the possibility of sample hemolysis. Working on colorectal cancer, Maierthaler et al. [67] reported on the dependency of plasma miRNA levels on the time-point of blood sampling and on hemolysis status. The time-point of blood sampling in relation to treatment was identified as a factor that strongly influenced the amounts of most circulating miRNAs analyzed in their validation cohort. Additionally, hemolysis influenced the levels of investigated miRNAs. Interaction analysis between miRNA expression and hemolytic status with respect to patient prognosis and survival revealed that miR-200a showed a strong interaction between hemolysis and prognosis within the metastatic patient group [67].

As regards response to chemotherapy, it seems that miR-200c overexpression increases in resonse to [41, 43] microtubule-targeting agents, such as paclitaxel in particular. This is strongly stated in the study by Brovozic et al. [47], where treatment with miR-200c and miR-141 mimics, although conferring resistance to carboplatin, sensitized the tumor cells to paclitaxel, presumably through the downregulation of β-tubulin III. Similarly, in other types of cancer, miR-200c overexpression enhances sensitivity to chemotherapy. Ma et al. [74] found that miR-200c overexpression inhibited the chemoresistance, invasion and colony formation of human pancreatic cancer stem cells. Knezevic et al. [75] reported that the induced expression of miR-200c in breast cancer cells with a low claudin expression significantly enhanced chemosensitivity and decreased the metastatic potential of this high-metastasizing tumor. Finally, Chang et al. [76] demonstrated that the loss of miR-200c upregulated CYP1B1 and conferred docetaxel resistance in renal cell carcinoma. Therefore, it seems possible that a high miR-200c and miR-141 expression is associated with decreased β-tubulin III expression, and a favorable responsiveness to paclitaxel chemotherapy, verifying miR-200 family members as regulators of EMT.

## CONCLUSIONS

The miR-200 family appears to play a significant role in OC. The present review examined the potential usefulness of miR-200 family members as prognostic indicators in OC. Specific points of literature review were addressed. First, miR-200c overexpression correlated with a favorable prognosis and could be viewed as a potential prognostic biomarker of OC. Second, miR-200 up-regulation (mainly miR-200a and miR-200c) seems to correlate with specific histological subtypes of OC. Third, high miR-200c and miR-141 expression seems to be associated with a decreased β-tubulin III expression and a favorable responsiveness to paclitaxel chemotherapy, verifying miR-200 family members as regulators of EMT. Finally, various miRNA signatures have been developed for disease prediction. However, the clinical application of all these points remains to be determined; therefore, further studies are warranted in order to explore the fundamental role of the miR-200 family in the initiation and progression of OC.
